# An Evaluation of a Borided Layer Formed on Ti-6Al-4V Alloy by Means of SMAT and Low-Temperature Boriding

**DOI:** 10.3390/ma9120993

**Published:** 2016-12-08

**Authors:** Quantong Yao, Jian Sun, Yuzhu Fu, Weiping Tong, Hui Zhang

**Affiliations:** 1 Key Laboratory of Electromagnetic Processing of Materials, Ministry of Education, Northeastern University, Shenyang 110819, Liaoning, China; yaoquantong@gmail.com (Q.Y.); fyzh1224@gmail.com (Y.F.); hzhang@epm.neu.edu.cn (H.Z.); 2 Department of Materials Science and Engineering, Hefei University of Technology, Hefei 230009, Anhui, China

**Keywords:** Ti-6Al-4V alloy, SMAT, low-temperature boriding, hardness, toughness

## Abstract

In this paper, a nanocrystalline surface layer without impurities was fabricated on Ti-6Al-4V alloy by means of surface mechanical attrition treatment (SMAT). The grain size in the nanocrystalline layer is about 10 nm and grain morphology displays a random crystallographic orientation distribution. Subsequently, the low-temperature boriding behaviors (at 600 °C) of the SMAT sample, including the phase composition, microstructure, micro-hardness, and brittleness, were investigated in comparison with those of coarse-grained sample borided at 1100 °C. The results showed that the boriding kinetics could be significantly enhanced by SMAT, resulting in the formation of a nano-structured boride layers on Ti-6Al-4V alloy at lower temperature. Compared to the coarse-grained boriding sample, the SMAT boriding sample exhibits a similar hardness value, but improved surface toughness. The satisfactory surface toughness may be attributed to the boriding treatment that was carried out at lower temperature.

## 1. Introduction

Titanium and its alloys are widely used in artificial bone implants, aircraft manufacturing, and kitchenware due to their excellent chemical and physical properties, such as high strength-to-weight ratio, good biocompatibility, and low corrosion/oxidation rate [1,2,3,4]. However, the poor wear resistance extensively limits their further applications [5,6]. Among various surface treatments, boriding is a kind of thermo-chemical treatment which can generate a hard borided layer on the surface of titanium alloys. Although the borided layer presents low thickness, it effectively enables the improvement of the tribological performance of titanium and its alloys [6]. Nevertheless, because of the low boron atomic diffusivity, the boriding process is conventionally performed at 1000 °C ± 100 °C for a long duration time, which may induce the formation of a large amount of porosity in the borided layer, resulting in the deterioration of surface toughness. The borided layer, with high brittleness, may bring some negative effects for the further application of titanium materials. Therefore, reducing the boriding temperature of titanium alloys has become a hot topic in the thermo-chemical treatment field.

It is well known that the nano-grained materials are characterized by a large number of nanocrystalline boundaries and dislocation substructures, which can act as fast atomic diffusion pathways [7]. Therefore, inducing a nanocrystalline layer on the surface of metallic materials seems to be an effective method to enhance the atomic diffusion kinetics, leading to a reduction in the thermo-chemical treatment temperature. In past decades, surface mechanical attrition treatment (SMAT) has been an effective method that enables coarse grains to refine into nanoscale grains at the surface of various metallic materials [8,9,10,11]. Previously, studies have shown that the nitriding, chromizing, and boriding of various metallic materials could be performed at lower temperature with the assistance of SMAT [12,13,14,15]. For example, T. Balusamy showed that SMATed EN8 steel can be borided with a reasonable case depth at 650 °C for 7 h [16]. Xu et al. have shown that boriding of SMATed H13 steel could be achieved at 650 °C for 8 h [17].

In this paper, we employed one of the most widely used Ti-6Al-4V alloys to study the low-temperature pack boriding behavior (600 °C) by assisting with a nanocrystalline layer. The phase composition, microstructure, and micro-hardness of the SMAT borided sample were studied by using X-ray diffraction (XRD), scanning electron microscopy (SEM), and transmission electron microscopy (TEM). In particular, the brittleness of the surface layer on the borided SMAT sample was investigated in comparison with that of coarse-grained sample borided at 1100 °C.

## 2. Results and Discussion

### 2.1. Microstructure Characterizations of the SMAT Sample

**Figure 1:**
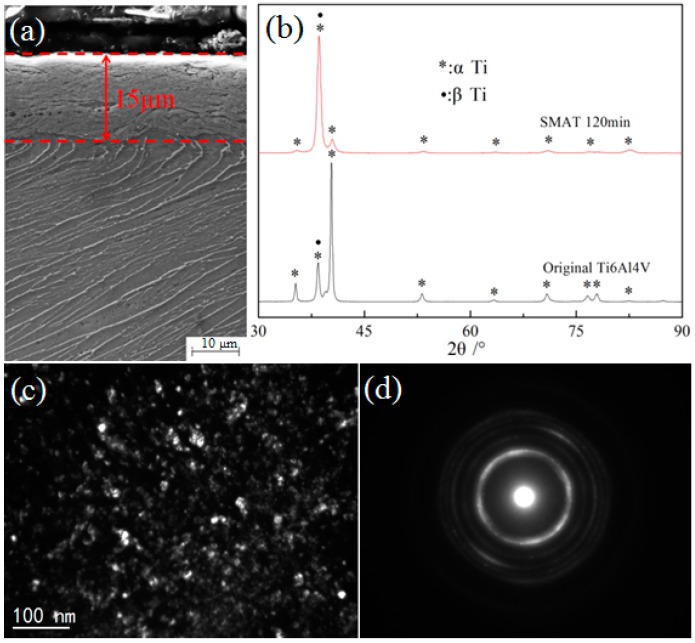
The microstructure characterizations of the SMAT Ti-6Al-4V alloy: (**a**) SEM micrographs; (**b**) XRD patterns; (**c**) TEM image (dark field); (**d**) SAED pattern.

**Figure 2:**
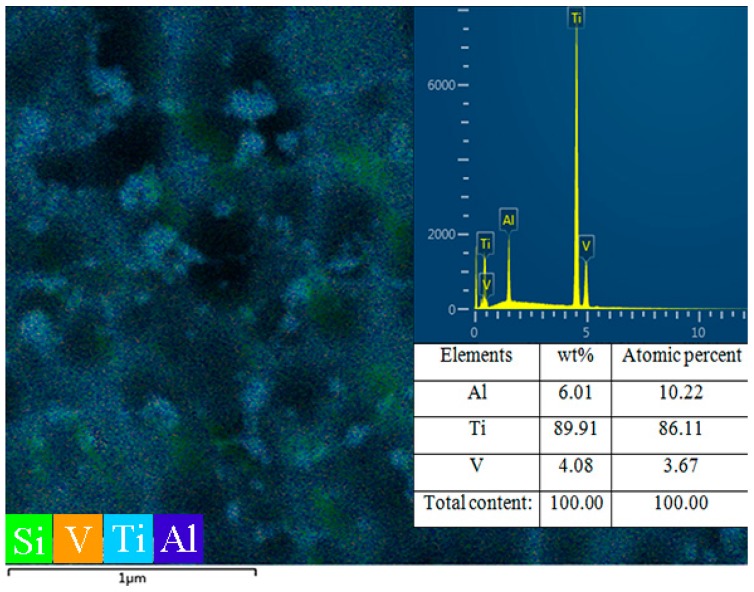
The EDS analysis of the sample surface after 120 min SMAT.

Figure 1 shows the microstructure characterizations of SMAT Ti-6Al-4V alloy by using SEM, XRD and TEM. It can be seen that an obvious deformation layer of 15 μm thickness was distinguished from the matrix, as shown in Figure 1a. The grain boundaries and microstructure are no longer clearly identified by SEM observation in the deformation layer. Figure 1b shows XRD patterns of the Ti-6Al-4V alloy before and after SMAT. An evident broadening of the Bragg reflections was found on the SMAT sample by comparing the full width at half maximum (FWHM), which may be attributed to the grain refinement and micro-strain development. Additionally, the broadening rate of Bragg reflections of α-Ti is more evident than that of β-Ti. Generally, dual-phase structural Ti-6Al-4V alloy coexists in the hexagonal-closed-packed (HCP) α-Ti structure and face-centred-cubic (FCC) β-Ti structure. Since the stacking fault energy is different between the α-Ti (HCP) and the β-Ti (FCC), the plastic deformation more easily occurs in the lower stacking fault energy structure of α-Ti (HCP) [18]. The similar results can be also observed by surface nanocrystallization process of dual/multi-phase structural alloy in other literatures [19,20,21]. There is another variation that should be paid attention to: the solid-state transformation (α-Ti→β-Ti) induced by SMAT. This can be attributed to the increase in strain energy, which may account for the earlier-stated structural instability due to the Gibbs–Thompson effect [22]. Thus, this structural instability due to grain size reduction and strain may ultimately cause a polymorphic change from α-Ti (HCP)→β-Ti (FCC), which has a lower ΔG or higher structural stability [23]. Further TEM observation and corresponding selected area electron diffraction (SAED) illustrated that the microstructure of the uppermost-treated surface layer is characterized by ultrafine, equiaxed grains with random crystallographic orientations, as shown in Figure 1c,d. The average grain size in the top surface layer is approximately 10 nm. The surface chemical composition of the SMAT sample was also detected by EDS in Figure 2. No oxides and contaminants could be detected in the SMAT sample, owing to the fact that we used the Ti-6Al-4V alloy as the container and impacting balls during the SMAT process.

### 2.2. Thermal Stability of SMAT Ti-6Al-4V

**Figure 3:**
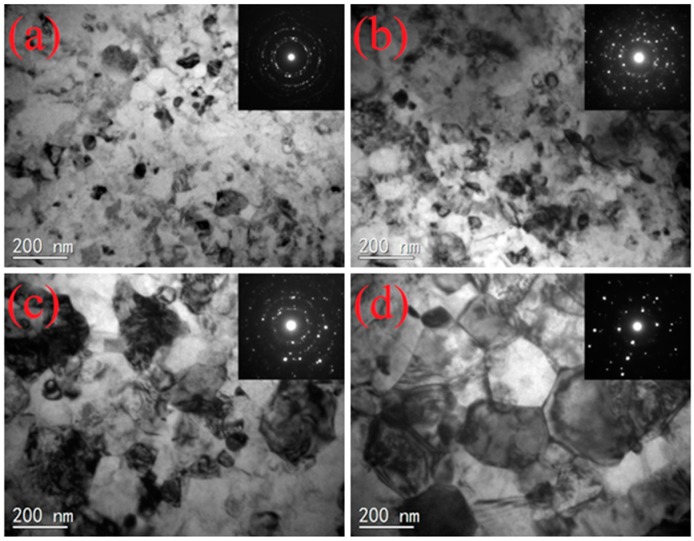
TEM images and corresponding SAED patterns of 120 min SMAT sample annealed for 5 h at different temperatures: (**a**) 550 °C; (**b**) 600 °C; (**c**) 650 °C; and (**d**) 700 °C.

In order to obtain a suitable pack boriding temperature, the thermal stability of the nano-grain was investigated by isothermal anneal for 5 h at various temperatures in a vacuum furnace. The effect of the annealing temperature on grain size was studied by TEM. As shown in Figure 3a,b, the nano-grain is still at the nano-scale (<100 nm) when the annealing temperature is at 550 °C and 600 °C. With the annealing temperature increased to 650 °C, a large number of sub-micron scale grains can be found on the surface layer, as shown in Figure 3c. Additionally, the grain size is about 100~300 nm. Meanwhile, corresponding SAED patterns present a discontinuous circular distribution, indicating that grain size no longer belongs to the nanoscale. When the annealing temperature increases to 700 °C, the grain size is about 300~500 nm, as shown in Figure 3d. According to the above TEM observations, the temperature of obvious abnormal grain growth could be confirmed at a range of 600~650 °C. Considering that the boron atoms’ diffusion in the nanocrystalline layer are very fast, which may hinder the growth of nanoscale grains to some extent, a boriding temperature of 600 °C (5 h) should be chosen in the following boriding experiment.

### 2.3. Microstructure Characterization of the Borided Layer

**Figure 4:**
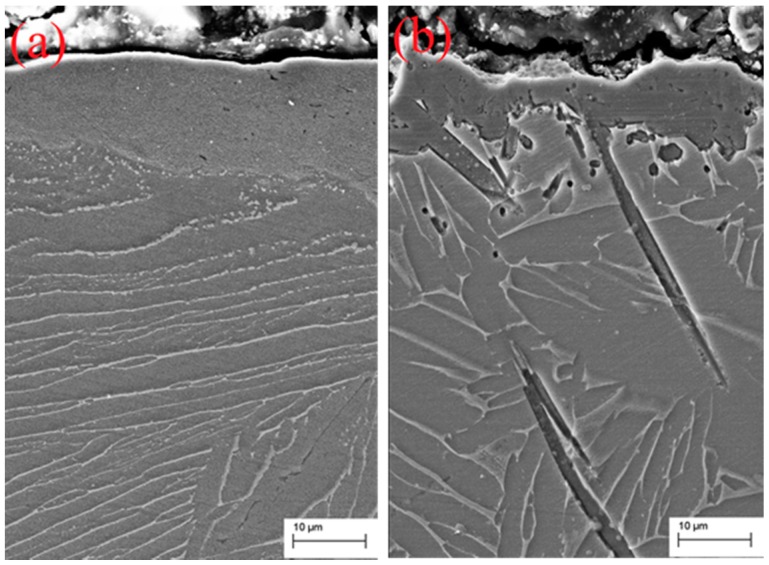
Cross-sectional SEM micrographs of the borided layer of (**a**) the SMAT sample and (**b**) the coarse-grained sample.

**Figure 5:**
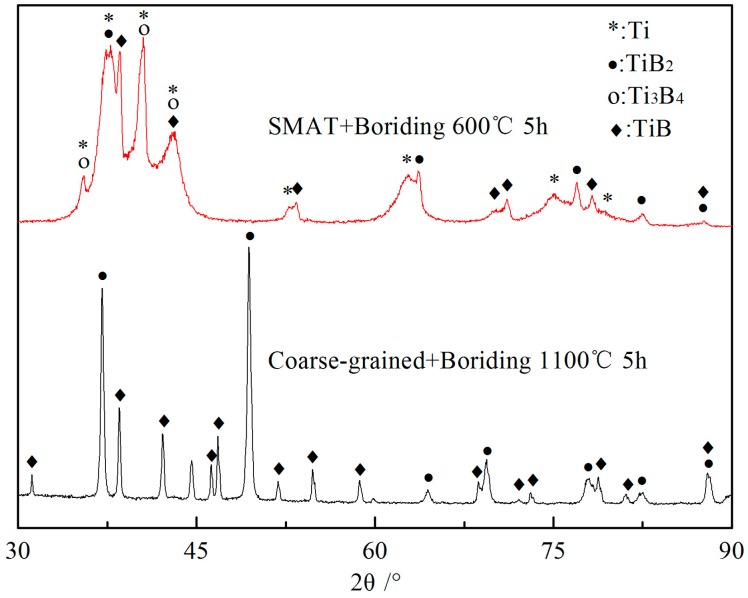
XRD patterns of the borided layer of the SMAT sample and coarse-grained sample.

**Figure 6:**
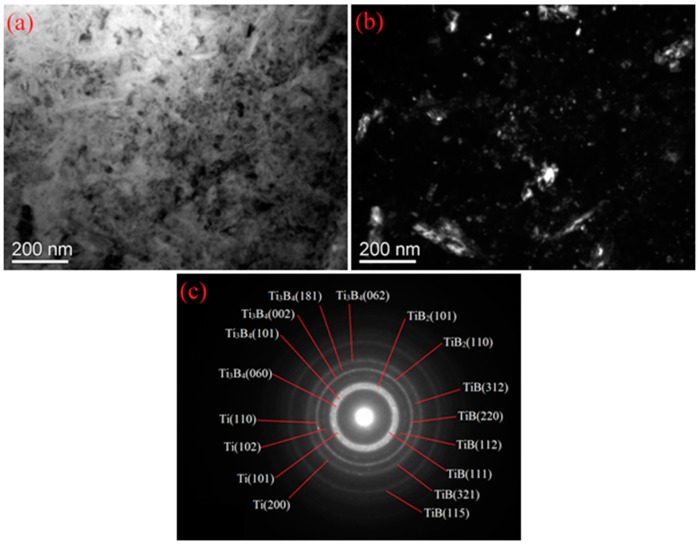
TEM images and corresponding SAED patterns of the borided layer of the 120 min SMAT sample: (**a**) light field; (**b**) dark field; (**c**) electron diffraction patterns.

Figure 4 shows the cross-sectional SEM micrographs of a SMAT sample borided at 600 °C for 5 h and a coarse-grained sample borided at 1100 °C for 5 h. Borided layers in the SMAT sample and coarse-grained sample present completely different morphology. A gray borided layer with a thickness of 15 μm was found on the surface of the borided SMAT sample, which is obviously thicker than that formed on the borided coarse-grained sample. Additionally, the borided layer of the coarse-grained sample presents a tooth-shaped morphology which extends into the sample interior. Similar tooth-shaped crystal whiskers cannot be found in the borided SMAT sample. In addition, the borided layer of the SMAT sample is discontinuous, indicating that the diffusion of boron atoms in the surface of the SMAT sample is mostly along the nanocrystalline boundaries and other crystallographic defects. Figure 5 shows XRD patterns of the borided layer of the two kinds of samples. The XRD patterns provide phase informations of about 20 μm thickness from the outermost surface to the interior. The Bragg diffraction peaks of the borided SMAT sample present obvious broadening after boriding treatment, indicating that the borides are in the nano-scale. This result can be attributed to the nanocrystalline remaining in the nano-scale without obviously increasing at 600 °C for 5 h. Therefore, the borides of the SMAT sample could be obtained in the nano-scale in the following boriding process. TEM images and their corresponding SAED patterns of the borided layer of the SMAT sample are presented in Figure 6. It can be seen that the borided layer consists of nano-scaled borides (20~30 nm). Additionally, the borides are composed of Ti, TiB, TiB_2_, and Ti_3_B_4_ by measuring the SAED patterns. These results are in agreement with the XRD analysis. The Ti_3_B_4_ is detected on the surface of the SMAT sample, but it cannot be detected on the coarse-grained sample. This result can be attributed to the Ti_3_B_4_ that was decomposing into TiB and TiB_2_ at a high boriding temperature of 1100 °C. Additionally, oxides are not detected on the surface of the SMAT sample and the coarse-grained sample from XRD and SAED analysis. 

### 2.4. Hardness of the Nitrided Layer

**Figure 7:**
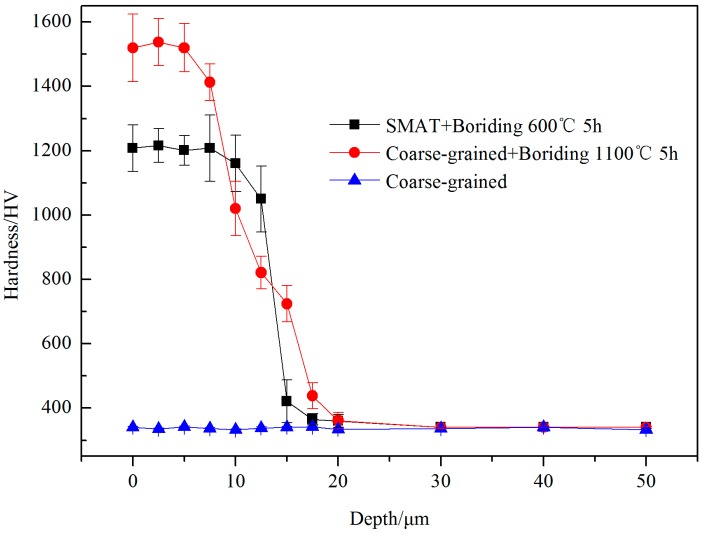
Hardness variations along the depth of both the SMAT boriding sample and the coarse-grained boriding sample.

In Figure 7, the micro-hardness of various samples along the cross-sectional direction from the outermost surface to the interior was measured by a micro-hardness tester. The micro-hardness of the coarse-grained sample is 340 HV by repeated measurement. The hardness of the SMAT sample is 550 HV, which is 1.6 times higher than that of the matrix. The hardness of the SMAT sample further increased to 1210 HV following the boriding treatment. Such a high hardness value is generally achieved at a higher temperature for a longer duration time in conventional boriding treatments [24]. Since the improved hardness is mainly caused by boride formation and boron atom diffusion, the thickness of the hardening layer can be considered as the depth of the boron atoms’ diffusion. As shown in the SMAT+Boriding curve, the hardening layer concentrates within 20 μm which is the range of the nanocrystalline layer. This means that boron atom diffusion was mainly along nanocrystalline boundaries. The boron atoms cannot diffuse into the crystal lattice of the matrix below the nanocrystalline layer at a low temperature of 600 °C. As shown in the coarse-grained+Boriding curve, the borided layer of coarse-grained sample presents a striking contrast with that of the SMAT sample. Although the coarse-grained sample is borided at the high temperature of 1100 °C, the effective thickness of the hardening layer is obviously thinner than that of the coarse-grained sample. The hardness of the outermost surface achieved 1520 HV, but it rapidly decreases within 20 μm. This result can be attributed to the low diffusivity of boron atoms in the Ti-6Al-4V alloy. Therefore, the boriding kinetics are effectively enhanced by nanocrystalline layer assistance.

### 2.5. Toughness of the Borided Layer

**Figure 8:**
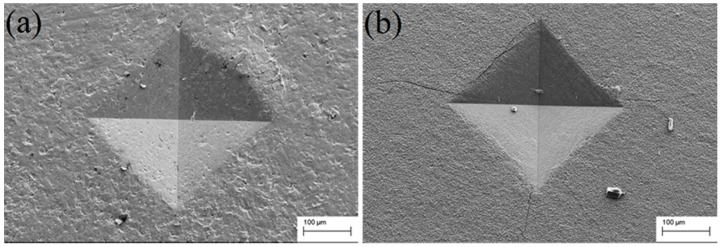
SEM micrographs of the Vickers indentation of both (**a**) the SMAT boriding sample and (**b**) the coarse-grained boriding sample.

In order to evaluate the toughness of the borided layer, the sharp indentation measurement method was utilized. The sharp indenters (conic or pyramidal) are normally used for the analysis of the toughness in opaque ceramic materials because the contact pressure is independent of the indentation size, and failure propagates from the corners of the residual impression [25]. The material toughness is established by three experimental parameters: the borided layer thickness, the indentation impression size, and the applied load [26]. Before the measurement, the surfaces of the various samples were ultrasonically cleaned in acetone for 15 min to remove the contaminants attached to the sample surfaces. The cleaned surfaces were immediately subjected to the Vickers hardness test using a load of 30 kg. Generally, there are two types of brittle crack modes from Vickers indentations: Palmqvist cracking mode and radial-median cracking mode. After the measurement, markedly different indentation morphologies of various samples are presented in Figure 8. It seems that the Palmqvist cracking mode is the main crack type in sample surfaces after boriding treatment. The main difference is that no obvious Palmqvist cracking can be identified from Figure 8a. This result means that the surface toughness of the SMAT samples has no obviously increase after boriding treatment. However, in sharp contrast with the SMAT sample, the coarse-grained sample in Figure 8b presents marked Palmqvist cracking after the boriding treatment. The numerous Palmqvist cracking originates from the angle position of the pyramidal Vickers indentation and extends to the surroundings. Thus, it can be seen that the boriding process decreases the toughness of the Ti-6Al-4V sample, whereas the SMAT sample maintains the toughness of the Ti-6Al-4V sample by nanocrystalline layer assistance.

## 3. Materials and Methods

Ti-6Al-4V cylinders with 49 mm in diameter, having a chemical composition of (in wt.%) 5.5~6.75, Al; 3.5~4.5, V; 0.3, Fe; 0.08, C; 0.05, N; 0.015, H; 0.2, O; and the remaining balance, Ti, were used in the present study. Firstly, the Ti-6Al-4V cylinders were annealed at 600 °C for 10 h to relieve the residual stress. Secondly, the Ti-6Al-4V cylinders were cut into sheets with a thickness of 3 mm by wire cut electrical discharge machining (WEDM). Thirdly, Ti-6Al-4V sheets were polished by 60 #~1000 # abrasive papers to remove the WEDM marks. Finally, Ti-6Al-4V sheets were ultrasonically cleaned in acetone for 10 min.

**Figure 9:**
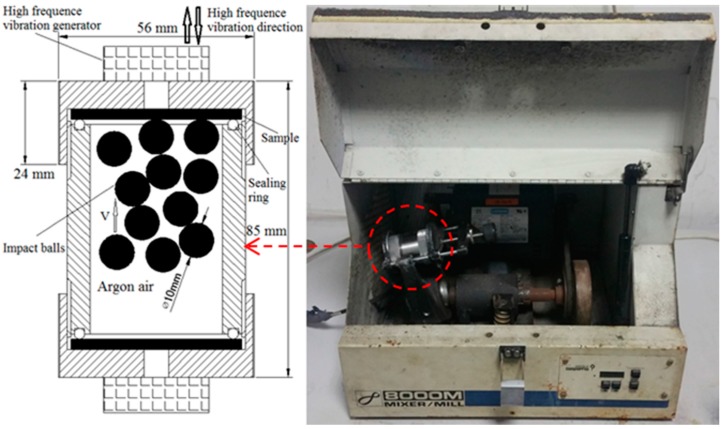
A schematic view of the Spex mill.

The SMAT process was performed by using a SPEX/8000M mill (SPEX^®^ SamplePrep, Metuchen, NJ, USA) which is shown in Figure 9. The Spex mills are widely used tools for synthesizing nanocomposites. Since they have the ability to provide vibration at high energy, they have an obvious potential to input sustained stress onto a sample surface, which induces the grains to refine to the nano-scale. There are a great number of studies that describe the Spex mill used in surface nanocrystallization process [7,12,27]. In order to prevent the incorporation of iron pollution products into the sample surface, the cylindrical container of the Spex mill was made from Ti-6Al-4V alloy. The cleaned Ti-6Al-4V alloy sheet was held in place via mechanical locking at one end of the cylindrical container of the Spex mill, and 20 Ti-6Al-4V alloy balls 10 mm in diameter were used to provide the desired impact on the surface of the Ti-6Al-4V alloy sheet. The impact veloctiy of Ti-6Al-4V balls induced by shaking the cylindrical container of the Spex mill was 15 m/s. In order to prevent the sample from oxidizing, the SMAT process was conducted under an argon atomsphere.

The SMAT sample was ultrasonically cleaned in acetone for 5 min, then immediately subjected to seal by a stainless steel container for the pack boriding process with B4C powder media (99.9 wt.%, 320 mesh). Additionally, the boriding process was performed in a muffle furnace at 600 °C for 5 h. For comparison, the coarse-grained sample was pack borided at 1100 °C for 5 h. After boriding treatment, the boriding container cooled down in the furnace.

The grain size, micro-strain, and phase information of nanocrystalline layer and borided layer were obtained with X’ Pert Pro PW3040/60 X-ray diffractometer (PANalytical, Lelyweg, EA Almelo, The Netherlands), (XRD) using Cu Kα radiation (40 kV, 40 mA). Small angular steps of 2θ = 0.03° were taken to measure the intensity of each Bragg diffraction peak. The grain size and micro-strain were derived from the breadth at half maximum intensity of measured Bragg diffraction peaks by using the Scherrer-Wilson equation [12]. Cross-sectional morphologies of various samples were observed by using a Zeiss Ultra 55 scanning electron microscope (Zeiss, Jena, Freistaat Thüringen, Germany) (SEM). Phase information of outermost surface layer of borided samples were characterized by a TECNAI G20 transmission electron microscope (FEI, Hillsboro, OR, USA) (TEM) from their corresponding selected area electron diffraction (SAED) patterns. The TEM samples were ground and mechanically polished, followed by ion thinning at a lower temperature.

The micro-hardness values along the surface to interior were measured with a Wolpert L101 MVD Vickers micro-hardness tester (Buehler, Lake Bluff, IL, USA) with a load of 25 g and a duration time of 10 s. Micro-hardness values were calculated by the measured geometry of the Vickers pyramid import. The surface toughness was evaluated by a Wolpert 450 SVA Vickers macro-hardness tester (Buehler, Lake Bluff, IL, USA) with a load of 30 kg and duration time of 10 s. The pyramidal indentation was characterized by using the Zeiss Ultra 55 SEM.

## 4. Conclusions

A surface nanocrystalline layer with a thickness of about 15 μm was fabricated on the surface of Ti-6Al-4V alloy after 120 min SMAT. The average grain size of the outermost surface was refined to about 10 nm with a random crystallographic orientation. The thermal stability of nanocrystalline could be maintained below 650 °C.

The low-temperature boriding kinetics of the SMAT sample was significantly enhanced by nanocrystalline layer assistance. The borided layer of the SMAT sample was composed of TiB, TiB2, Ti3B4, and Ti with supersaturated boron atoms.

Compared to the coarse-grained borided sample, the SMAT borided sample exhibits a similar hardness value, but improved surface toughness. Meanwhile, the obvious brittleness crack was not found on the borided layer of the SMAT sample. The excellent toughness of borided layer of the SMAT sample may be attributed to the low boriding temperature.
